# Introducing an adolescent cognitive maturity index

**DOI:** 10.3389/fpsyg.2022.1017317

**Published:** 2022-12-07

**Authors:** Shady El Damaty, Valerie L. Darcey, Goldie A. McQuaid, Giorgia Picci, Maria Stoianova, Veronica Mucciarone, Yewon Chun, Marissa L. Laws, Victor Campano, Kinney Van Hecke, Mary Ryan, Emma Jane Rose, Diana H. Fishbein, Ashley S. VanMeter

**Affiliations:** ^1^Interdisciplinary Program in Neuroscience, Georgetown University, Washington, DC, United States; ^2^Center for Functional & Molecular Imaging, Georgetown University Medical Center, Department of Neurology, Washington, DC, United States; ^3^Institute for Human Neuroscience, Boys Town National Research Hospital, Boys Town, NE, United States; ^4^Center for Pediatric Brain Health, Boys Town National Research Hospital, Boys Town, NE, United States; ^5^Department of Psychology, The Pennsylvania State University, University Park, PA, United States; ^6^Translational Neuro-Prevention Research, Frank Porter Graham Child Development Institute, University of Northern Carolina, Chapel Hill, NC, United States

**Keywords:** adolescence, cognitive development, age prediction, maturity, dual systems model, regularized regression, structural equation model

## Abstract

Children show substantial variation in the rate of physical, cognitive, and social maturation as they traverse adolescence and enter adulthood. Differences in developmental paths are thought to underlie individual differences in later life outcomes, however, there remains a lack of consensus on the normative trajectory of cognitive maturation in adolescence. To address this problem, we derive a Cognitive Maturity Index (CMI), to estimate the difference between chronological and cognitive age predicted with latent factor estimates of inhibitory control, risky decision-making and emotional processing measured with standard neuropsychological instruments. One hundred and forty-one children from the Adolescent Development Study (ADS) were followed longitudinally across three time points from ages 11–14, 13–16, and 14–18. Age prediction with latent factor estimates of cognitive skills approximated age within ±10 months (*r* = 0.71). Males in advanced puberty displayed lower cognitive maturity relative to peers of the same age; manifesting as weaker inhibitory control, greater risk-taking, desensitization to negative affect, and poor recognition of positive affect.

## 1. Introduction

### 1.1. Estimating cognitive age in developing adolescents

The transition from adolescence to emerging adulthood is a fuzzy boundary with no standard demarcation that can be applied to every child. Currently, there are no cheap, non-invasive, and accessible tools for tracking the individual growth curves of developing teenagers. The adolescent brain goes through significant changes during the approach to adulthood, suggesting that cognitive skills may be promising markers for tracking maturation (Shulman et al., 2016). Skills such as active maintenance of goal-related representations in working memory, inhibitory control of reflexive behavior, weighing of risks vs. rewards, and processing emotional context have been identified as key developmental traits leading to adulthood (Luna et al., 2015). Mainstream models of adolescent brain development underscore emotional reinforcers as key drivers for social learning and cognitive control skills (Jones et al., 2014; Rosenbaum et al., 2020). These models are corroborated by behavioral and neuroimaging evidence suggesting that cognitive skills, emotional processing, and the brain regions that support them, develop together during adolescence. However, the timing of neurocognitive skill maturation and the interaction between cognitive control, risk-taking, and emotion processing is not yet fully elucidated (Duell et al., 2016; Shulman et al., 2016). Although existing models of adolescent cognitive development have been helpful for understanding the transition to adulthood, a key missing aspect is a data-driven operational definition of neurocognitive age or maturity. A more precise definition has the potential to help distinguish sources of variation in development and help translate science for utilitarian social applications by identifying critical developmental windows during which particular interventions may exert the greatest benefits (Somerville, 2016). For example, variance in standardized testing scoring curves could be adjusted for cognitive skills maturity to aid personalized assessment of educational level and course placement. Tailored cognitive skills training may yield more persistent and effective results if delivered during key periods of developmental plasticity (Knoll et al., 2016). Adolescent decision making is heavily biased by social and environmental context. Identification of individual growth curves may reveal periods of enhanced vulnerability to adverse outcomes and may provide opportunities for intervention (Kirisci et al., 2009). Early biological models of age based on DNA methylation, transcription and telomere length (Baker and Sprott, 1988; Jylhävä et al., 2017), brain structure (Khundrakpam et al., 2015; Aycheh et al., 2018; Madan and Kensinger, 2018), and brain network oxygen metabolism (Dosenbach et al., 2010; Qin et al., 2015) have exhibited significant success in predicting age and identifying individual developmental trajectories bench-marked against the average growth curve in the sampled population. However, cognitive age prediction using theory-driven indicator variables obtained from behavioral experiments has yet to be implemented. Cognitive age prediction with neuropyschological assessments would be cheaper, less invasive, and more accessible to researchers and practitioners compared to biometric assays.

### 1.2. Improving on the model-free approach

No complete model of the biological mechanisms of age-related change exists yet, and so exploratory research has relied on statistical techniques that compress a large number of features to extrapolate sample-specific effects (Crimmins et al., 2008; Sagers et al., 2020). Common methods for age prediction typically use big-data-driven approaches, involving the collection of large amounts of data per individual, such as genome-wide RNA transcription, or MRI measurements analyzed using hundreds of thousands of pair-wise correlations between voxels at discrete time intervals. These methods pose an issue in that there are significantly more descriptive features per individual than there are samples in the dataset (i.e., “the curse of dimensionality”; *p*>>*n*, Taylor, 2019)—a problem that commonly leads to overfitting with standard linear regression. This issue is overcome through the application of data reduction and variable selection techniques, such as principal component analysis or regularized regression, that penalize or eliminate redundant features (Lee and Yoon, 2017). Model-free techniques have been successful for predicting age within an acceptable error range (Cole and Franke, 2017); however, these models do not always lend themselves to interpretation because variable selection and data reduction can be biased by their cost function, leading to overfitting (Babyak, 2004), or confounded by collinear indicators of age, such as motion in fMRI experiments (Satterthwaite et al., 2013).

### 1.3. Modeling maturity with latent variables of behavior

A potential remedy for the curse of dimensionality in age prediction is specifying models with data derived from experiments designed to isolate and measure features of age-related change. A hierarchical approach that involves (1) identifying key cognitive metrics, (2) their interactions, and (3) finally correlations with behavior enhances statistical power and the interpretability of tested models. Currently, there are no applications utilizing behavioral tasks developed for tracking age-related change with the goal of estimating cognitive age in typically developing adolescents. Here, we describe a method for computing a cognitive maturity index (CMI) using reaction time, task performance, and other derived metrics from the Continuous Performance (CPT), Wheel of Fortune (WOF), Emotional Face Recognition (EFR), and Temporal Delay Discounting (TD) tasks collected in the Adolescent Development Study (Fishbein et al., 2016). First, confirmatory Factor Analysis (CFA) is used to estimate directly unobservable latent cognitive factors predicted to change with age during adolescence; such as inhibitory control, risk/reward processing, and emotion recognition. The interaction between factors is tested in a structural equation model (SEM) and latent factor estimates of cognitive skills used as predictors in a regularized regression model to predict age. The CMI is defined as the difference between observed and predicted cognitive age for each participant. A high CMI indicates accelerated cognitive maturity relative to the sample mean, whereas a low CMI indicates a relatively lagging developmental trajectory. This work demonstrates that predicting cognitive age using latent constructs is a promising technique that can be scaled with larger neurocognitive datasets to generate more accurate population estimates for adolescent neurocognitive maturity and further illuminate the interaction between social context and trajectories of neurocognitive development.

## 2. Methods

### 2.1. Participants

Early adolescent youth were recruited to participate in the Adolescent Development Study (ADS), a prospective, longitudinal investigation of the neurodevelopmental factors underlying early substance use initiation and the consequences on brain development (Fishbein et al., 2016). Youth (*N* = 141) from the Washington, D.C. Metro area were enrolled in 2011 (Table 1). As of March 2020, participants had completed up to four sequential waves of data collection separated by approximately 18 months. Eligibility at wave 1 included the following criteria: (1) ages 11–13 years, (2) right-handedness, (3) no history of neuropsychiatric disorders or recent head injuries, (4) no self-reported consumption of one or more units of alcohol or other substances and (5) not of direct Asian descent. Asian participants were excluded at the onset of the study to control for genetic metabolic differences in the dose-response curve to alcohol. A total of six participants were excluded following the baseline visit due to: substance use at baseline (*N* = 2), autism diagnosis (*N* = 1), and high scores on ambidexterity measured with the Edinburgh Handedness Test (*N* = 3) (Veale, 2014). Despite attrition, the distribution of sex and race remained approximately the same throughout the study (±0.5%). Participants were compensated and reimbursed for travel, when applicable. All youth and caregivers gave their informed assent and consent prior to data collection and study procedures were reviewed and approved by the Georgetown University Institutional Review Board.

**Table 1:** Demographic, risk, and neuropyschological indicators assessed across development.

		**Wave one**	**Wave two**	**Wave three**	***P*** **(>||χ||)**
Sex					0.930
	Female	65 (53.60%)	62 (54.50%)	60 (54.0%)	
	Male	57 (46.40%)	54 (45.5%)	48 (46.0%)	
Race					0.918
	White	65 (54.60%)	62 (55.40%)	61 (58.10%)	
	Black	37 (31.10%)	35 (31.20%)	29 (27.60%)	
	Hispanic	8 (6.70%)	5 (4.50%)	4 (3.80%)	
	Other	9 (7.60%)	10 (8.90%)	11 (10.05%)	
Age					< 0.001
	Mean (SD)	12.68 (0.76)	14.32 (0.82)	15.84 (0.80)	
	Range	11.11–14.00	12.41–16.12	13.87–18.01	
BMI					0.004
	Mean (SD)	21.01 (4.73)	22.00 (5.04)	23.14 (5.15)	
	Range	14.40–45.46	15.35–47.90	15.59–46.17	
Composite IQ					0.380
	Mean (SD)	110.66 (14.25)	108.03 (15.26)	109.02 (13.13)	
	Range	75.00–139.00	72.00–136.00	84.00–138.00	
DUSI-VP					< 0.001
	Mean (SD)	2.79 (2.05)	3.53 (2.50)	4.23 (2.76)	
	Range	0.00–9.00	0.00–10.00	0.00–11.00	
BIS					0.395
	Mean (SD)	20.12 (3.30)	20.34 (3.67)	20.78 (3.92)	
	Range	12.00–28.00	13.00–27.00	10.00–28.00	
BAS-D					0.012
	Mean (SD)	9.83 (2.55)	10.18 (2.42)	10.81 (2.37)	
	Range	4.00–16.00	5.00–16.00	5.00–16.00	
BAS-FS					0.509
	Mean (SD)	11.50 (2.39)	11.23 (2.25)	11.16 (2.29)	
	Range	4.00–16.00	5.00–16.00	6.00–16.00	
BAS-RR					0.849
	Mean (SD)	17.68 (1.67)	17.61 (1.82)	17.54 (1.97)	
	Range	14.00–20.00	12.00–20.00	13.00–20.00	

χ^2^ test revealed significant effect of Age, BMI, DUSI-VP, and BAS-D across waves. A total of 23 unique participants were excluded from summary statistics at any wave due to high DUSI-LIE.

### 2.2. Interview procedure and collected metrics

Pre-screened, eligible participants were invited for an on-site visit at Georgetown University Medical Center to complete a series of questionnaires and interviews designed to measure neurocognitive developmental traits and capture social and family life. The accompanying primary caregiver was interviewed in a separate room and asked to complete a questionnaire regarding economic status, education, and the difficulty of acquiring basic needs such as food, healthcare, and housing (Bornstein et al., 2003). Socioeconomic Status (SES) was estimated from these responses by converting the family's household income to z-scores, averaging both guardian/parents' years of education, converting the average to a z-score, and lastly averaging the income and education z-scores for the final SES measure (Manuck et al., 2010).

#### 2.2.1. Anthropometrics

Adolescents had their height and weight measured and completed the Pubertal Development Scale (Carskadon and Acebo, 1993) to measure body-mass index (BMI) and physical changes with age at each wave. Pubertal development scores were derived from an interview that recorded self-reported changes in height, body hair, complexion, vocal pitch, breast size, and menarche. Respondents responded with: “has not yet begun,” “has barely begun,” “is definitely underway,” or “is complete” for each puberty related physical features queried by the instrument.

#### 2.2.2. Instruments

All participants were assessed for verbal and performance IQ using the Kaufman Brief Intelligence Test (KBIT) (Kaufman, 2004) and completed The Behavioral Inhibition System/Behavioral Activation System (BIS/BAS) Scale to provide a measure of appetitive and avoidant behavioral tendencies (Carver and White, 1994). The BAS is divided into three subscales measuring funseeking (BAS-FS; four items, ex. “I will often do things for no other reason than that they might be fun.”), independent drive (BAS-D; four items, ex. “I go out of my way to get things I want”) and reward responsivity (BAS-RR; five items, ex. “When I get something I want, I feel excited and energized”) used to measure self-reported idiosyncratic differences in temperament and personality underlying reinforcement sensitivity (Corr, 2004). Participants were asked on a four-point scale how well a particular statement characterized them (1:strongly agree to 4:strongly disagree).

### 2.3. Neurocognitive tasks

Participants completed the Continuous Performance and Wheel of Fortune tasks while undergoing functional MRI (Siemens Tim Trio 3T Scanner) during waves one through three. All participants were trained on the tasks outside of the scanner before entering the scanning room. Stimuli were projected onto a screen and reflected into the participant's field of view using a mirror. Participants responded using fiber optical button boxes. The Emotional Face Recognition and Temporal Discounting tasks were completed outside of the scanner on a laptop in a private behavioral testing room in the first three waves. The Facial Emotion Recognition Task was administered with E-Prime 1.2. All other neurocognitive tasks were built and presented with the E-prime Stimulus Presentation Software Version 2.0 (Schneider et al., 2002). Previous findings with these tasks have been published, validating associations between neural activity, impulsivity, and risk-taking (Darcey et al., 2019, 2020; Trojanowski et al., 2021).

#### 2.3.1. Continuous performance task (CPT)

The continuous performance task was used to measure impulsivity and inhibitory control of reflexive actions (Horn et al., 2003). Participants viewed five blocks of 30 letters presented one-at-a-time for a 200 ms duration (150 total trials). Each block was separated by a “cool-down” period in which a gray fixation cross was presented for 1, 300 ms. Participants were instructed to press the right-hand button box as quickly as possible for all letters except “Q”. The lure “Q” appeared 27 times in the task. The sequence of letters was the same across all participants. Signal Detection Theory metrics were utilized for analyzing CPT behavior (Stanislaw and Todorov, 1999). “Hits” were defined as correct button presses to target letters and “Misses” as failure to respond to a target letter. “False-Alarms” were defined as an incorrect response to the lure “Q”, whereas “Correct Rejections” were defined as correct inhibition of response. Discriminative sensitivity to lures was measured by the *d*′ variable, *d*′ = ϕ^−1^(*Hits*)−ϕ^−1^(*FalseAlarms*), and response-bias calculated by utilizing the natural log-transformed beta estimate, β = 0.5*[ϕ^−1^(*FalseAlarms*)^2^−ϕ^−1^(*Hits*)^2^]; where ϕ^−1^ is the inverse probability distribution function (Forbes et al., 2011).

#### 2.3.2. Wheel of fortune task (WOF)

A modified version of the Wheel of Fortune (WOF) task was administered to test propensity of risk-taking and gambling strategies (Ernst et al., 2004). The task was divided into three runs of 30 trials. In each trial, a “wheel” appeared on the screen that portrayed the probabilities of winning different amounts of virtual money. Participants were instructed to select between large monetary gains with a low probability (high-risk) or small monetary gains with high probability (low-risk) and indicated their choice using button boxes placed in their left and right hands (corresponding to choice of the left and right sides of the wheel, respectively). Winning probability varied pseudo-randomly between a 10:90% split (occurring 32 to 42 times of a total 90 trials) and a 30:70% split (occurring 48 to 58 times). The quantities of money assigned to the left and right side of the wheel varied between a $1–$9 for the low risk choice or a $2–$18 split for the high risk choice, when the wheel was split 10:90. The quantities similarly varied between $3–$7 and a $9–$21 split when the wheel was divided 30:70. These values and proportional assignments assured that the expected value (EV) appeared equal for a winning selection independent of risk (e.g., 30% chance of winning $7=$2.10 EV vs. 70% chance of winning $3= $2.10 EV). Spatial position of the rewards varied evenly, with the larger reward appearing on each side 50% of the time. The wheel was visible until the participant made their selection, or for a maximum of 3, 000 ms, followed by a 3, 000 ms delay after which feedback was presented indicating whether the participant had won or lost the selected dollar amount, along with their cumulative winnings up to and including that trial. Participants automatically lost the higher dollar amount if no decision was made before 3, 000 ms had elapsed. Each run began with a 6, 000 ms fixation, and the inter-trial interval was varied based on a Poisson distribution between 2, 500 and 10, 000 ms. The total quantity of money won or lost would be reset to $0 at the beginning of each run. Participants were encouraged to improve upon the amount they won in the next run. Participants were encouraged to respond as if their gains and losses were real, however no real money or physical reward was given. Task performance was analyzed using the probability of high vs. low risk decisions, the reaction time to make those decisions and the cumulative winnings at each run. Anticipatory responses were defined as trials <200 ms reaction time and discarded from analysis as outliers. The Wheel of Fortune Task has been implemented outside of the scanner and validated as a reliable predictor of real life risky behavior (Rao et al., 2011).

#### 2.3.3. Emotional face recognition task (EFR)

Participants viewed 70 photographs (grayscale images, 284 x 351 pixels, resolution = 96 dpi) from the NimStim dataset, which includes images of 29 professional actors aged 21–30 years (12 female, 17 male, 14 European-American, 10 African-American, 3 Asian-American 2 Latino-American) instructed to pose for expressions of seven emotions: happiness, surprise, sadness, anger, disgust, fear, and neutral (Tottenham et al., 2009). To prepare for the task, participants were presented showcards with labels of each of seven emotions and asked to describe each of the emotions followed by a practice trial for each emotion. For each trial, a photograph appeared for a maximum duration of 5, 000 ms along with seven labels for each of the emotions. Participants were instructed to click on the emotion with a mouse to advance to the next trial. The image disappeared after 5, 000 ms and labels remained until a response was made. Trials were separated by an inter-trial interval of 3, 000 ms, during which time participants viewed a white screen. No actor was shown with the same emotion more than once. The accuracy and reaction time for disgust, anger, sadness and fear were averaged together to compose estimates of performance for recognizing negative emotion. Positive emotion recognition performance was derived from reaction time and accuracy for happy faces only.

#### 2.3.4. Temporal delay discounting task (TD)

Individual preference between small, immediate vs. large, delayed rewards was tested by offering participants a forced choice between two options: “Would you rather have $*X* now or $*X*+*Y* in *Z* days?” The delay of rewards, *Z*, was varied from 0, 1, 2, 10 days, 1 month, half a year, and 1 year. The immediately available amount, *X*, was determined using a random adjustment procedure that updated the current choice based on previous choices. Immediate reward amounts varied from $0.50 to $10 while the delayed reward value *X*+*Y* was fixed at $10. The propensity for discounting the objective value due to delayed receipt was computed as the area under the curve (AUC) using the trapezoidal method: $AUC=\sum_{t=0}^{t=k}\left(d_{t+1}-d_{t}\right)*\left(v_{t}+v_{t+1}/2\right)$, where *t* is the current normalized delay point, *k* the maximum delay and *v* the indifference value at that point (Myerson et al., 2001; Olson et al., 2007; Borges et al., 2016). Indifference points reflecting the subjective value of the immediately available reward at a given delay were normalized to the maximum award value of $10 and plotted across time delays normalized to a duration of a year (annualized). As described above, trapezoids formed by normalized subjective values at each normalized delay contributed to AUC in the range from 0 to 1. A large AUC indicates participants are more likely to prefer large but delayed rewards whereas a lower AUC indicates preference for immediate gratification. In order to elicit behavior reflective of the participant's actual preferences, participants were informed that they would receive either a $5 or $10 reward based randomly on their choices in the task prior to the task.

### 2.4. Statistical analysis

Data were converted from double-entered paper records, Qualtrics survey exports, and E-prime task outputs then consolidated into a single long-format data frame containing an observation for each participant at each wave. Next, descriptive statistics and assessments of multivariate normality were performed with the Arsenal (3.5.0), MVN (5.8, Korkmaz et al., 2014), and DescTools (0.99.36, Signorell, 2020) packages in R version Orange Blossom (1.2.5033, R Core Team, 2019). Participants with high DUSI-R Lie scores (*N* = 23; Lie>6) were excluded from analysis (Dalla-Déa et al., 2003). Demographics for the excluded participants did not significantly differ from the retained group. Scored neurocognitive and sociodemographic measures were correlated with age to identify and confirm expected bivariate relationships with development, including that of age-related changes in neurocognitive skills as revealed by EFR, CPT, WoF, TD task performance. Longitudinal effects of task performance by age were performed using the lmer R package to account for hierarchies of repeated measures in multi-level models (Bates et al., 2015). Task performance at each wave of data collection was modeled as a nested factor within participants and used to estimate the average intercept and rate of change across all participants and within subgroups controlling for random effects attributable to individual differences. First, we assessed the Confirmatory Factor Analysis (CFA) was implemented with the Lavaan R package (0.6–6) to estimate latent factors for inhibitory control (CPT), risk/reward processing (WOF/TD), and emotional face recognition (EFR) (Rosseel, 2012). Latent factor analysis is a statistical method for estimating an underlying mechanism that can only be indirectly measured through indicators derived from experimental constructs (Finch and French, 2015). Latent factor estimates are obtained by averaging the unique contribution of each indicator variable after controlling for the shared variance across indicators to satisfy the local independence principle (Sobel, 1997). Maximum likelihood estimation with full information maximum likelihood (FIML) was used to adjust CFA parameter estimates for missing data (Cham et al., 2017). The latent factors were standardized to allow for free estimation of factor loadings and *post hoc* testing (Hu and Bentler, 1998). The model fit of the implied structural relationships was assessed with χ^2^ Goodness of Fit referenced against a null model with 0 factor loadings, the Root Mean Square Error of Approximation (RMSEA), the Comparative Fit Index (CFI) and the Tucker-Lewis Index (TLI; Hu and Bentler, 1999; Wu et al., 2009; Kenny et al., 2015). Path and structural models were visualized with the semPlot R package (1.1.2). The standardized latent factor estimates of inhibitory control, risk-taking and facial emotion recognition were used to predict age with regularized linear regression models implemented in the glmnet R package (4.0-2) using leave-one-out cross-validation for estimation of model hyperparameters and a 50% random split across participants between training and test datasets (Friedman et al., 2009, 2010). Cross-validated model performance was estimated by computing the *R*^2^ from the mean cross-validated error divided by the variance of observed age in the test sample across the regularization rate (λ) and penalty factor (α; 0=Ridge Regression, 0.5= Elastic Net, 1= Least Absolute Shrinkage and Selection Operator) hyper-parameter estimates. The minimum λ at the highest *R*^2^ was used to calculate regression coefficients in the training sample. The cognitive maturity index was estimated as the difference in the observed and predicted age for each participant.

## 3. Results

### 3.1. Latent factor analysis of cognitive metrics

#### 3.1.1. Inhibitory control

**Figure 1:**
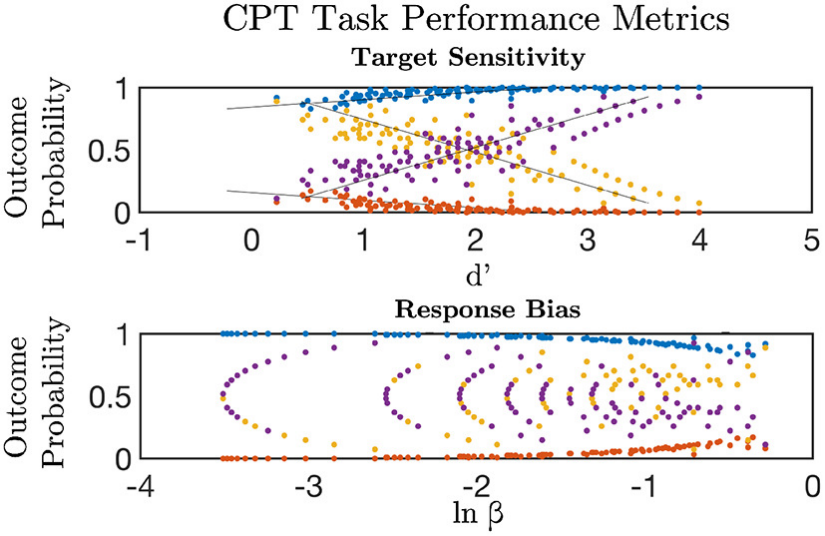
Signal detection theory metrics were used to estimate discriminative sensitivity (d') and overall response bias (ln β) for targets versus lures in the Continuous Performance Task (CPT). d' increased linearly with higher probability of a Correct Rejection (purple) and response to target (Hits, blue); and declined with greater False Alarm responses (yellow) and Miss rate (red) to targets (top). The probability of a Correct Rejection and response to target was a non-linear decreasing function of increasing response bias (bottom). Greater false alarm rates were indicative of elevated response bias and lower target-lure discrimination.

The ICLF was estimated using the CPT response time standard deviation, log transformed response bias, *ln*(β), target discrimination, *d*′, and the BIS part of the BIS/BAS. The fitted relations were significant compared to a null model with 0 factor loadings between all measures, residual covariances, and the latent variable estimate (Table 2; *CLI* = 1.0; *TLI* = 1.018; *RMSEA* < 0.01, *p* = 0.91). Greater discriminative ability between false alarms and targets was significantly inversely correlated to response bias (−0.46 ± 0.14; *Z* = 3.36; *p* = 0.001) and was associated with greater variation in response time to targets (0.30 ± 0.09; *Z* = 3.36; *p* = 0.001) (Figure 1). This was corroborated by a strong linear relationship between *d*′ and the average response time to targets (Supplementary Figure 4; 0.57 ± 0.05; *Z* = 12.11, *p* < 0.001). Response time variance to targets interacted inversely with false alarm response time to predict *d*′ (−0.08 ± 0.02; *Z* = −3.35, *p* < 0.001). Response time variation was uncorrelated between target and false alarm trials (0.07 ± 0.09; *Z* = 0.70, *p* = 0.48) demonstrating that each provided a statistically independent contribution to ICLF. The ICLF increased with greater age (0.72 ± 0.01; *Z* = 7.50, *p* < 0.001). The ICLF did not vary by sex, or BMI, but was found to significantly increase with SES (0.267 ± 0.053; *Z* = 4.99, *p* < 0.001) and pubertal development (0.14 ± 0.06; *Z* = 2.22, *p* = 0.03).

**Table 2:** Normalized estimates for latent factors estimated with structural equation modeling of inhibitory control using continuous performance task metrics and the behavioral inhibition system scale.

**Inhibitory control latent factor**	**Estimate**	**Std. error**	***Z*** **-value**	***P*** **(>||*z*||)**
CPT target discrimination (d')	0.650	0.090	7.189	< 0.001
CPT response bias (β)	−0.503	0.124	−4.059	< 0.001
CPT hit RT standard deviation	−0.913	0.115	−7.910	< 0.001
CPT false alarm RT standard deviation	−0.371	0.085	−4.367	< 0.001
Behavioral inhibition system (BIS)	0.193	0.058	3.335	0.001

CFI = 1.00, TLI = 1.018, RMSEA = 0.001, *p* = 0.910.

#### 3.1.2. Risk-taking

The RRLF was estimated using probability of high risk decisions, cumulative winnings and response time in the WOF task, and area under the curve of TD performance. Overall, RRLF was characterized by greater risky decisions, moderate response time for high risk options, longer response time for low risk decisions, significantly poorer cumulative winnings and stronger propensity for immediate rewards in the TD task (Table 3). RRLF significantly decreased with age (−0.22 ± 0.10; *Z* = −2.35, *p* = 0.019). The model fit to the data significantly improved by allowing free estimation of covariance between the probability of making a high risk decision and response time, and between low and high risk response times. The fitted relations with these free parameters revealed a significant fit of the covariance structure compared to a null model with 0 factor loadings between all measures, residual covariances, and the RRLF estimate (*CLI* = 1.0; *TLI* = 1.0; *RMSEA* = 0.005, *p* = 0.846). The estimate of the covariance between response time and the probability of making a high risk decision revealed that risky decisions occurred more quickly than carefully evaluated low risk options (−0.121 ± 0.031; *Z* = −3.849, *p* < 0.001). Overall, the RRLF was related to greater risk-taking in the WOF task, which resulted in poor cumulative winnings due to high probability of loss and consequently running a negative balance. RRLF did not significantly covary with sex, PDS, BMI, or SES.

**Table 3:** Normalized estimates for latent factors estimated with confirmatory factor analysis summarizing reward/risk-taking in the Wheel of Fortune Gambling and Temporal Delay Discounting tasks.

**Reward/risk latent factor**	**Estimate**	**Std. error**	***Z*** **-value**	***P*** **(>||*z*||)**
WOF percent high risk choices	0.703	0.087	8.106	< 0.001
WOF high risk mean reaction time	0.158	0.060	2.648	0.008
WOF low risk mean reaction time	0.433	0.073	5.971	< 0.001
WOF cumulative winnings	−0.899	0.076	−11.817	< 0.001
Temporal discounting	−0.127	0.064	−1.974	0.048

CFI = 1.00, TLI = 1.00, RMSEA = 0.005, *p* = 0.846.

#### 3.1.3. Emotional face recognition

Performance in the EFR task was used to derive latent factors of positive and negative emotional face recognition (EPLF/ENLF). Latent factors were estimated using the mean and standard deviation of response time and accuracy to recognize angry, fearful, sad, or disgusted (negative) and happy (positive) facial expressions. The model fit was improved significantly by permitting free covariation between mean reaction time to negative and positive faces, negative and positive emotion recognition accuracy, and between standard deviation and mean response time for both negative and positive emotions. The proposed model structure explained a significant proportion of the covariance between task metrics and provided an excellent fit compared to a null model (Table 4; *CLI* = 0.978; *TLI* = 0.942; *RMSEA* = 0.073, *p* = 0.115). Perceptual processing of positive emotional faces often resulted in correct emotion recognition, but was associated with generally longer and more variant time to recognition, reflecting difficulty in distinguishing between happy and other facial expressions. Perceptual processing of negative emotional faces often resulted in correct recognition with quick and consistently low variation in recognition time. Perceptual processing performance for negative emotions was inversely related to positive emotion processing (standardized estimate −3.44 ± 1.85; *Z* = −1.86, *p* = 0.063). ENLF, but not EPLF, was found to increase with SES (0.16 ± 0.07; *Z* = 2.11, *p* = 0.036) and BMI (0.04 ± 0.02; *Z* = 2.65, *p* = 0.008). No significant relationship between ENLF and sex or PDS was revealed. Greater physical maturation measured with PDS was related to lower EPLF scores (−0.70 ± 0.15; *Z* = −4.55, *p* < 0.001). ENLF significantly increased with age (0.35 ± 0.14; *Z* = 2.44, *p* = 0.015) whereas EPLF decreased with age (−0.16 ± 0.10; *Z* = −1.52, *p* = 0.129). No relationship between EPLF and SES, BMI, or sex was found.

**Table 4:** Normalized estimates for latent factors estimated with confirmatory factor analysis summarizing emotional face recognition task performance for positive and negative emotions.

**Negative emotions latent factor**	**Estimate**	**Std. error**	***Z*** **-value**	***P*** **(>||*z*||)**
EFR accuracy	0.164	0.088	1.869	0.062
EFR mean reaction time	−0.451	0.128	−3.536	< 0.001
EFR standard deviation of reaction time	−0.302	0.085	−3.541	< 0.001
Positive emotions latent factor				
EFR accuracy	0.173	0.088	2.245	0.092
EFR mean reaction time	0.310	0.132	2.702	0.048
EFR standard deviation of reaction time	0.235	0.093	2.818	0.042

CFI = 0.978, TLI = 0.942, RMSEA = 0.073, *p* = 0.115.

### 3.2. Structural equation model of cognitive factors

**Figure 2:**
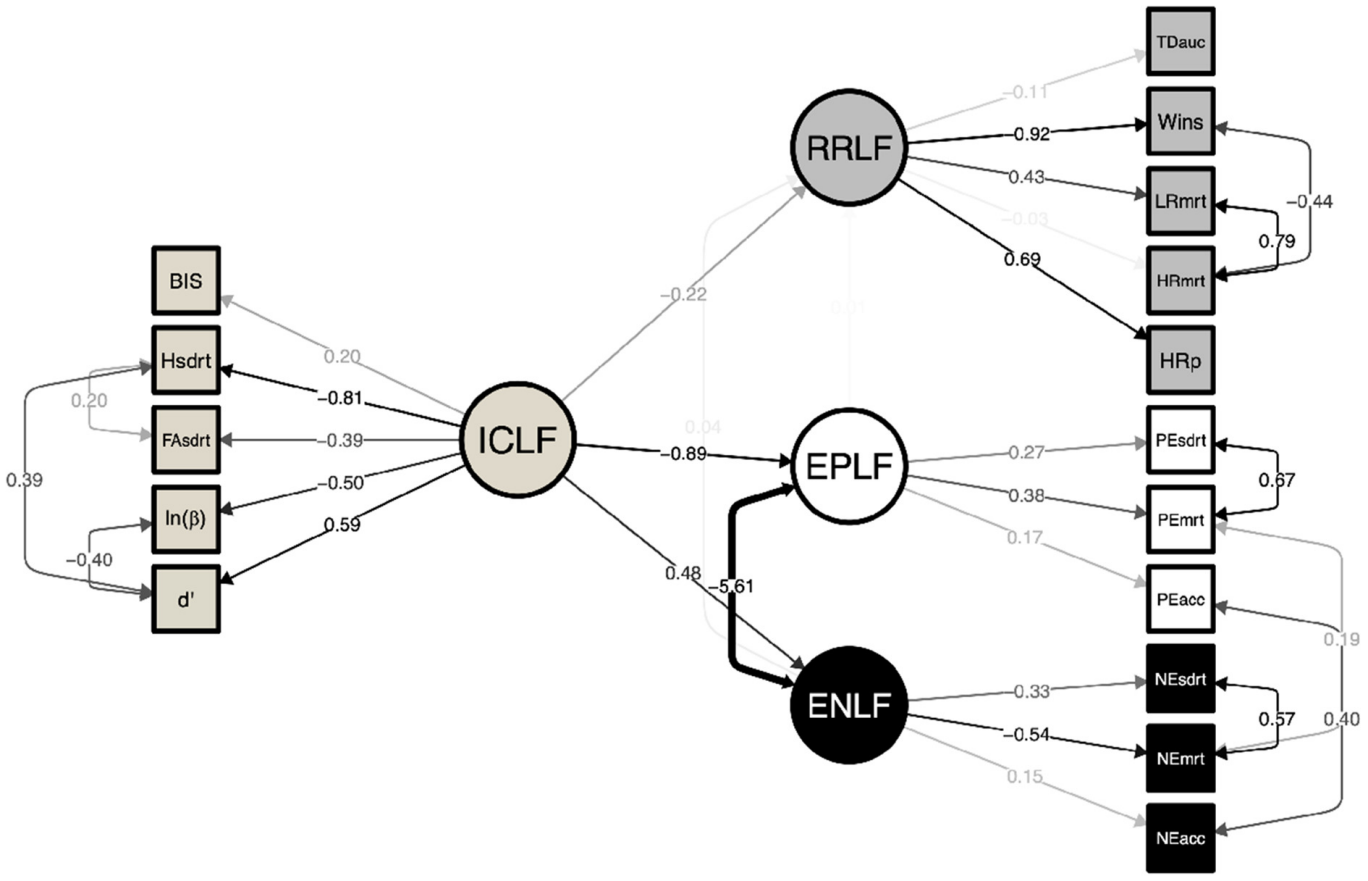
Structural equation model of latent factors underlying inhibitory control (ICLF), risky reward processing (RRLF), and responsivity to positively (EPLF) and negatively salient emotional faces (ENLF; RMSEA = 0.047, TLI = 0.926, CFI = 0.945). Inhibitory control was observed to be a significant effector of lower risk taking (*p* = 0.026) and responsivity to both negative (*p* = 0.007) and positive (*p* = 0.02) emotional faces. No significant relationship was observed between RRLF and EPLF/ENLF. The ENLF manifested as fast and accurate responses to negative emotions, whereas EPLF was an indicator of longer looking times leading to correct recognition. ICLF effected faster looking time to all emotions at the expense of recog- nizing happy face expressions. Paths are faded to indicate statistical significance and strength of association. Numerical edge labels provide standardized estimates.

A structural equation model was constructed to explore the interaction between the identified latent factors and their indicator variables (Figure 2). Significant covariations were identified between the constituent cognitive latent factors compared to a null model (*CLI* = 0.95; *TLI* = 0.93; *RMSEA* = 0.047, *p* = 0.64). ICLF was found to significantly reduce RRLF (−0.22 ± 0.10; *Z* = −2.16, *p* < 0.031). Greater inhibitory control also predicted greater sensitivity to negative emotional faces (0.55 ± 0.26; *Z* = 2.09, *p* = 0.037) and effected faster recognition time for all emotions at the expense of recognizing happy facial expressions. As previously noted, elevated ENLF was inversely correlated with EPLF. No relationship was found between RRLF and negative or positive emotional processing.

### 3.3. Cognitive maturity index

**Figure 3:**
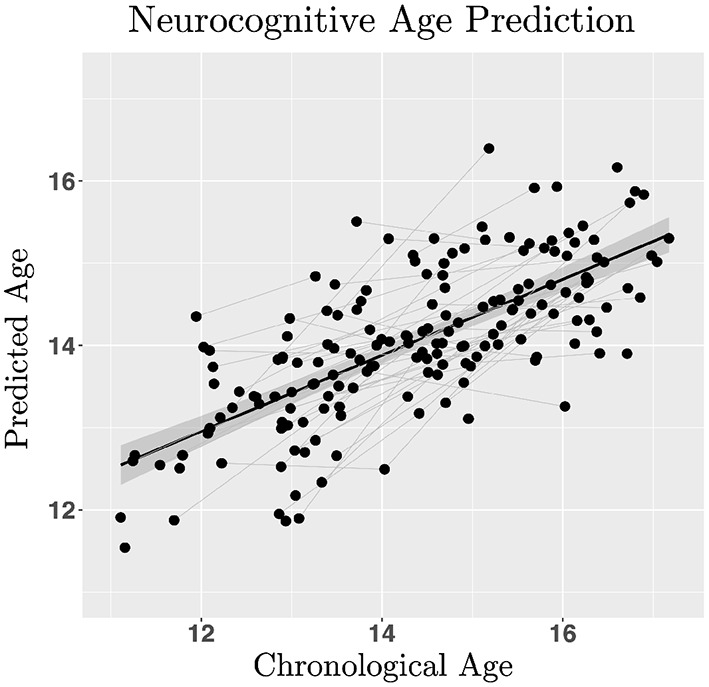
Regularized regression was used to estimate neurocognitive age in a training sample with leave-one-out cross validation to estimate linear model hyper- parameters (lambda = 0.083; L2-norm ridge regression with alpha = 0) minimizing mean squared error for predicting age with inhibitory control, risk/reward processing and emotional processing latent factors in a test sample (50% participants split into train/test datasets). Model performance was assessed by computing the ratio between the mean cross-validated error and variance of observed age in the validation dataset (R^2^ = 0.51). The neurocognitive maturity index is computed by subtracting the predicted neurocognitive age from the chronological age.

Linear ordinary least squares regression was used to test ICLF, RRLF, EPLF, and ENLF as predictors of chronological age to explore the utility of a predictive model of neurocognitive age. Inhibitory control (0.72 ± 0.01; *Z* = 7.50, *p* < 0.001) and negative affect perceptual processing (0.35 ± 0.14; *Z* = 2.44, *p* = 0.015) significantly increased in efficiency with age, whereas risky reward taking (−0.22 ± 0.10; *Z* = −2.35, *p* = 0.019) and positive affect recognition (−0.16 ± 0.10; *Z* = −1.52, *p* = 0.129) declined. Ridge regression (α = 0, λ = 0.083) with latent factors predicted age in a split-half test sample within a mean absolute error of ±10.11 months (*R*^2^ = 0.51; Figure 3), significantly more accurately than by training on the original indicator variables used to generate the latent variable estimates (*R*^2^ = 0.16). The difference between predicted and observed age, defined as the cognitive maturity index (CMI), was computed for each participant. Greater CMI correlated with lower BMI (Pearson's *R* = −0.24, *p* < 0.001), slower pubertal development (*R* = −0.20, *p* < 0.001), lower BAS-D (*R* = −0.16, *p* < 0.001), higher IQ scores (*R* = 0.20, *p* < 0.001), and lower DUSI-R problem scale scores on: substance use (*R* = −0.20, *p* < 0.001), health risk (*R* = −0.16, *p* < 0.001), and, lastly, risk for violence (*R* = −0.28, *p* < 0.001). No significant relationship was found between SES and CMI, or between any of the other BIS/BAS scales and CMI. Advanced pubertal development (−0.67 ± 0.089; *Z* = −7.47, *p* < 0.001) interacted with sex (−2.44 ± 0.39; *Z* = −6.21, *p* < 0.001) to predict greater CMI in more fully developed females compared to males (interaction effect estimate 0.82 ± 0.16; *Z* = 5.30, *p* < 0.001).

## 4. Discussion

The distinction between chronological and biological age has been explored in great detail, but not yet extended to account for different trajectories of neurocognitive maturation during adolescence. The current study demonstrates a novel method for identifying behavioral hallmarks of cognitive development that can be used to estimate within-sample maturity in adolescents. This technique and derived findings have significant implications for predicting adverse life outcomes in emerging adulthood, such as risk for substance use and experiences of violence. Reliable prediction of age-related adverse outcomes is a significant unlock for prevention practice, as it opens opportunistic windows for intervention and cognitive skills training (Lipsey et al., 2010; Dorn et al., 2019). Theory-driven variable reduction was implemented with regularized regression for optimized age-prediction accuracy (±10 months). This accuracy exceeds age prediction performance using expensive neuroimaging datasets (±1 year), which typically incorporate a massive number of features, leading to overfitting (Franke et al., 2012; Cole and Franke, 2017). Structural equation modeling revealed a significant interaction between inhibitory control ability with risk-taking and emotional face recognition. Where advanced inhibitory control skills correlated with tempered risk-taking and higher sensitivity to negative emotional faces. The interaction between inhibitory control, risk-taking, and sensitivity to emotional faces covaried with age, supporting claims that the development of cognitive control skills occurs with greater sensitivity to emotional stimuli during adolescence (Casey et al., 2019). Latent cognitive factors were used as predictors to reliably estimate chronological age in a validation sub-sample. The Cognitive Maturity Index was calculated as the residual between observed chronological age and predicted cognitive age and found to be a reliable predictor of cognitive skills maturity. A negative residual indicated a delay in cognitive skills development, whereas a positive residual indicated an advancement relative to the sample average. The CMI also reliably predicted life outcomes, such as IQ, risk for violence, and risk for substance use disorder. Implications, limitations, and connections of these findings to previous literature and future directions are discussed below.

### 4.1. Latent cognitive factors

In this study, latent cognitive factors underlying cognitive skills were estimated using a battery of neurocognitive tasks. Behavioral data collected during performance of the CPT, WOF, and EFR tasks were modeled with confirmatory factor analysis to reveal latent factors illustrating inhibitory control, risk/reward processing, and positive and negative emotion perceptual processing. Confirmatory factor analysis was a first step following a model-driven variable reduction procedure to estimate the weighted partial correlation of significant predictors related to specific cognitive skills. The resulting estimate was considered as a weighted combination of the residual covariance between indicator variables used to approximate the inferred latent factor, where the correlation between two indicator variables is permitted when they are both caused by the underlying latent variable (Cooper et al., 2019). This approach serves to reduce the number of variables required to model a hypothesized cognitive phenomenon and helps minimize the contribution of residual error on tests of statistical inference; both useful and necessary requirements for building predictive regression models that avoid over-fitting with collinear predictors.

#### 4.1.1. Inhibitory control skills

A high inhibitory control latent factor (ICLF) score was characterized by consistently careful responses in the CPT, along with overall behavioral caution measured by the BIS. The significant contribution of the BIS to the ICLF was also indicative of generic internalizing behavior and disposition toward caution, supporting previous findings (Malloy-Diniz et al., 2007; Loree et al., 2015). Careful responses in the CPT are reflected by greater proactive control of motor reflexes and careful discrimination between targets and lures. Discrimination was predicted by an inverse interaction between response time variance to targets and false alarm response time, indicating that a rapid response to early false alarms led to greater caution later in the task, resulting in overall improvement in target/lure discrimination and CPT performance (Supplementary Figure 1). This finding is reinforced by a significant inverse correlation between discrimination and both (1) a bias to respond to a stimulus, and (2) a greater response time variation to targets. The inverse correlation between bias and response time variation with discrimination suggests participants modified their behavior and responded with more consistent timing as they learned the task. Response time standard deviation was selected for the model *a priori* because mean reaction time did not significantly vary trial-by-trial across participants except at the beginning of a block. Additionally, participants were instructed to press the response button as fast as possible and variations in responses were typically only observed circa false alarm trials during which more cautious participants would modify their pace to avoid errors. Task performance improved from wave one to three independent of task learning effects (Supplementary Figures 2, 3). Inhibitory control did not covary by sex but was found to correlate positively with SES, suggesting that elevated social status may temper impulsive decision-making, supporting previous findings linking lower inhibitory control with lower SES (Spielberg et al., 2015). Lastly, advanced pubertal development also indicated greater ICLF scores, demonstrating a clear developmental effect related both to time and physical maturation.

#### 4.1.2. Risk and reward processing

The risk/reward evaluation latent factor (RRLF) was characterized by impulsive risk-taking and a preference for immediate rewards. Impulsive high risk decisions were preferred when perceived losses were greater and high risk was concurrent with high rewards. A higher RRLF score was indicative of longer deliberation times for low risk decisions compared to high risk decisions. This response bias toward high risk decisions is reinforced by a preference for immediate winnings revealed in the TD task. Risk/reward processing was expected to be inversely correlated with SES, but no significant relationship was found in the tested sample. Greater SES typically related to lower risk-taking on average within the sample, supporting previous findings (Holmes et al., 2019). No associations were found with sex, BMI, or pubertal development. A key missing component are real winnings as part of the WoF task. A higher stakes context may result in more nuanced risk-taking that breaks along SES categories.

#### 4.1.3. Emotion processing

Emotional face recognition performance was characterized with measures of reaction time and the correct identification of positive and negative emotions. Negative and positive emotional face processing were found to be inversely correlated—suggesting participants generally tuned to facial expressions of negative affect were more likely to exhibit rapid and inaccurate recognition of positive affect. Greater EPLF scores were reflected by longer and more variant reaction times, indicating that participants were more careful identifying positive compared to negative emotions. As with elevated inhibitory control, sensitivity to negative emotions was found to be significantly greater in higher SES youth. A positive association with ENLF and BMI suggested greater sensitivity to negative affect in youth with higher body mass. Furthermore, greater physical maturation measured with PDS was related to lower EPLF scores, indicating that physically mature youth exhibit faster, less variable, recognition time and poorer accuracy for happy facial expressions. No relationship between EPLF and SES, BMI, or sex was found. In summary, these results indicate that physically mature youth from more prosperous, more educated, households are more sensitive to recognizing facial expressions of negative affect but do not possess comparable performance on positive affect recognition.

#### 4.1.4. Cognitive skills interactions

Structural equation modeling of the interaction between cognitive latent factors showed elevated inhibitory control, resulted in lower risky decision making. This finding demonstrates that a greater degree of inhibitory control was reflected as a lower propensity for risk-taking. Inhibitory control was also correlated with greater negative emotion perceptual processing skill, corroborating that adolescent cognitive development is concurrent with a greater sensitivity to negative social reinforcers (such as negative emotional context, or in this study faces of disgust, anger or fear; Jones et al., 2014; Rosenbaum et al., 2020). This finding may also supports claims that cognitive skills development is facilitated by increased sensitivity to reinforcement signals in the form of expected positive or unexpected negative outcomes during social and general task learning (Jones et al., 2014; Rosenbaum et al., 2020). Social signals, such as facial expressions, have been demonstrated to attract automatic attention, modulate hedonia, and serve as reinforcers of socially desired behaviors (Speer et al., 2007; Teufel et al., 2009). Strong emotional context during social reinforcement learning has been shown to capture attention and increase the speed and accuracy of learning new associations (Roper et al., 2014; Vernetti et al., 2017). Taking these findings into account, cognitive maturity appears to be driven by sensitivity to emotional reinforcers, reduced risk-taking and strong inhibitory control skills. Overall, our approach demonstrates that latent factors underlying cognitive skills development in adolescence can be estimated with standard well-validated cognitive tasks and instruments. Structural equation modeling revealed significant interactions between latent cognitive factors, where inhibitory control is expected to increase monotonically with age while tempering risk-taking into adulthood. The results highlight the importance of including cognitive processing of emotion in models of adolescent brain development. A normative development of inhibitory control was revealed to be concordant with emotion recognition ability and sensitivity bias to perceptions of negative emotional faces. Socioeconomic status was a significant covariate of cognitive skills development and calls attention to the importance of attending to social environmental context in models of adolescent neurocognitive development.

#### 4.1.5. Individual differences in cognitive maturity

The Cognitive Maturity Index is an individual level estimate of cognitive skills development. This estimate is relative to the mean within-sample best-fitted linear growth curve. The CMI was derived from latent factor estimates of inhibitory control, risk-taking, reward processing, and emotional recognition skills. Latent factor analysis provided best estimates of cognitive control skills for building a predictive model of adolescent neurocognitive maturity compared to the use of standard behavioral metrics (i.e., response time, performance) for predicting age. Improved estimation is credited to the minimization of measurement error through enforcement of local independence during latent factor estimation.

Several complementary models of adolescent brain development describe adolescence as a period of mismatch between the efficiency of cognitive control and affective salience of reinforcers (Casey et al., 2008; Luna and Wright, 2016). This work finds supporting evidence that weaker inhibitory control interacts strongly with higher risk-taking and desensitization to emotional faces. Furthermore, individual differences in cognitive skills directly correlate with participant-level variation in maturity. Participants with greater inhibitory control, lower risk-taking, and sensitivity to negative emotions exhibited a higher CMI relative to their peers.

Greater CMI translated to higher IQ scores and lower risk for substance use, health problems, and chances of experiencing violence by adulthood. CMI and SES did not show a significant correlation, suggesting that social context is not a strong driver for developmental maturity and may be modulated by status-specific risk factors. For example, affluent youth have previously been shown to be more risk tolerant and reward sensitive (Luthar and Becker, 2002; Luthar, 2003). Although, greater SES was shown to be related to higher risk-taking and lower inhibitory control as measured by the latent factor estimates, social context does not appear to be a significant effector of observed maturation within the sample. In other words, individual differences may a show a significant effect of social status on specific cognitive skills but it does not translate to reliable differences in chronological vs. cognitive age. Sex and Pubertal development (PDS) were found to interact as significant effectors of cognitive maturation. Females exhibited earlier/faster pubertal development and accelerated cognitive maturation (i.e., greater inhibitory control, less risk-taking, emotion sensitization) on average. Males with a higher scale of physical development were more likely to score high on the BAS-D, a measure of tenacity and desire for achieving goals and receiving rewards. Higher BAS-D scores in males were related to a delayed CMI compared to their peers. This finding corresponds with evidence that estrogen facilitates cognitive development and plasticity in women (Hara et al., 2015). The interaction with physical development and lower cognitive maturity rates in men provide a novel window into associated sex-specific risk-factors. Physically developed teenage males are often expected to be more mature, because they are bigger and look like adults. However this work demonstrates that physically mature men are more likely to be cognitively delayed relative to their peers, placing them at greater risk associated with cultural perceptions of masculinity (Chu et al., 2005).

## 5. Conclusions

A lack of consensus regarding the dynamics of cognitive skills development and their effect on adverse outcomes in emerging adulthood is compounded by the difficulty of defining when adolescence ends and adulthood begins in regards to neurocognitive development. In this work, we apply open-source statistical methods to provide a simple first step for approximating individual-specific neurocognitive age with linear latent factor modeling of inhibitory control, risk-taking, and emotional face perceptual processing skills. This approach embraces the perspective that maturity is best modeled as a relative factor and occurs along a continuum well into adolescence and emerging adulthood. Here we demonstrate sample-relative estimates of maturity can be derived from behavioral performance on neurocognitive instruments. There are several limitations worth mentioning in this work. First off, the growth curves for cognitive skills were estimated with an ordinary least squares linear regression model. No quadratic effects were tested, meaning that non-linear growth curves could not be assessed. This was chosen because the study was performed only over three time points, the minimum required for estimating non-linear mixed effects (Bollen and Curran, 2006). The three time point limitation minimizes the chances of a significant goodness-of-fit for cubic or higher-order growth curves. Furthermore, the CMI is a normalized estimate computed at each wave relative to the mean expected age in the sample and thus is an indicator of maturity only in the context of the sampled population. Significantly greater sample sizes will be required to extrapolate population level growth curves. Secondly, the Wheel of Fortune and Continuous Performance tasks were performed within the scanner and may not generalize to out of scanner performance. Follow up work may include validating in- with out-of scanner performance for these tasks. This work provides a foundation for others to apply this method with larger datasets, such as the ABCD project (Casey et al., 2018; Volkow et al., 2018), to better approximate a population estimate of adolescent neurocognitive maturity. Fusion with neuroimaging data may also serve to augment estimates by including biological mechanisms, to better delineate interactions between environmental and biological contributions to cognitive skills development.

## Data availability statement

The raw data supporting the conclusions of this article will be made available by the authors, without undue reservation at https://osf.io/cdyxh/.

## Ethics statement

The studies involving human participants were reviewed and approved by Georgetown Institutional Review Board. Written informed consent to participate in this study was provided by the participants' legal guardian/next of kin.

## Author contributions

SE wrote the manuscript, analyzed the data, devised the analytic method for CMI, and collected data for wave four under mentorship from DF, ER, and AV. VD, GM, and VM collected waves one through three data and made significant contributions to the manuscript. VD was vital for scoring and interpreting the temporal delay discounting task. GM documented the facial emotion recognition task. MS contributed to the manuscript, was responsible for importing paper records to RedCap, and managed data automation efforts for this project. YC, KV, and MR contributed to the manuscript, data collection, and coding wave four data for analysis. ML and VC contributed to the manuscript, completed the coding, and also contributed to the analysis of the wheel of fortune task. GP made intellectual contributions to the manuscript, including revising and editing. All authors contributed to the article and approved the submitted version.
