# Supplementary figures and images for: Introducing an adolescent cognitive maturity index
Source: Front Psychol. 2022 Dec 7;13:1017317. doi: 10.3389/fpsyg.2022.1017317 (PMC9771453; doi:10.3389/fpsyg.2022.1017317)

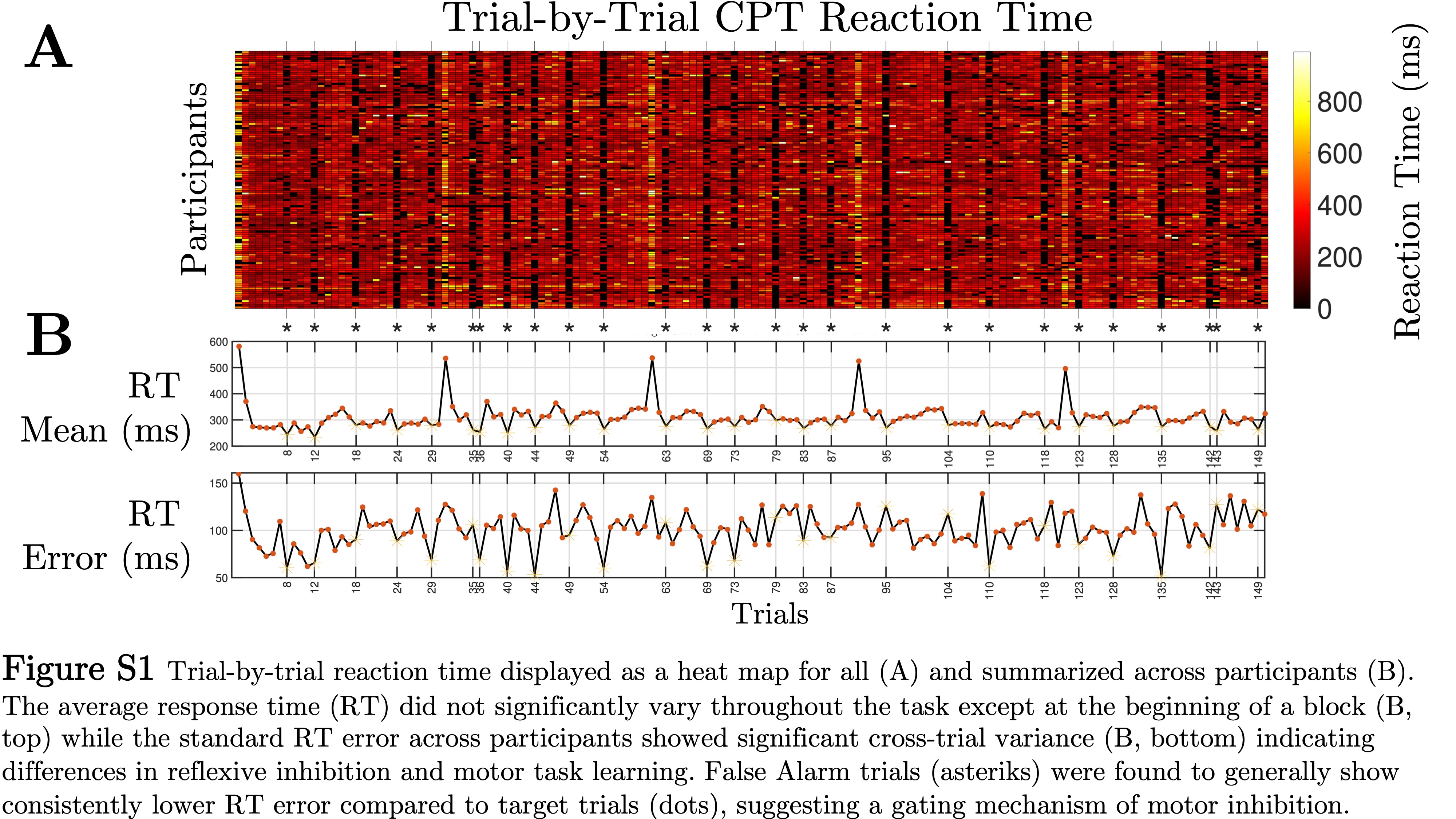

Supplement: Supplementary file 1 [file Image_1.jpeg]

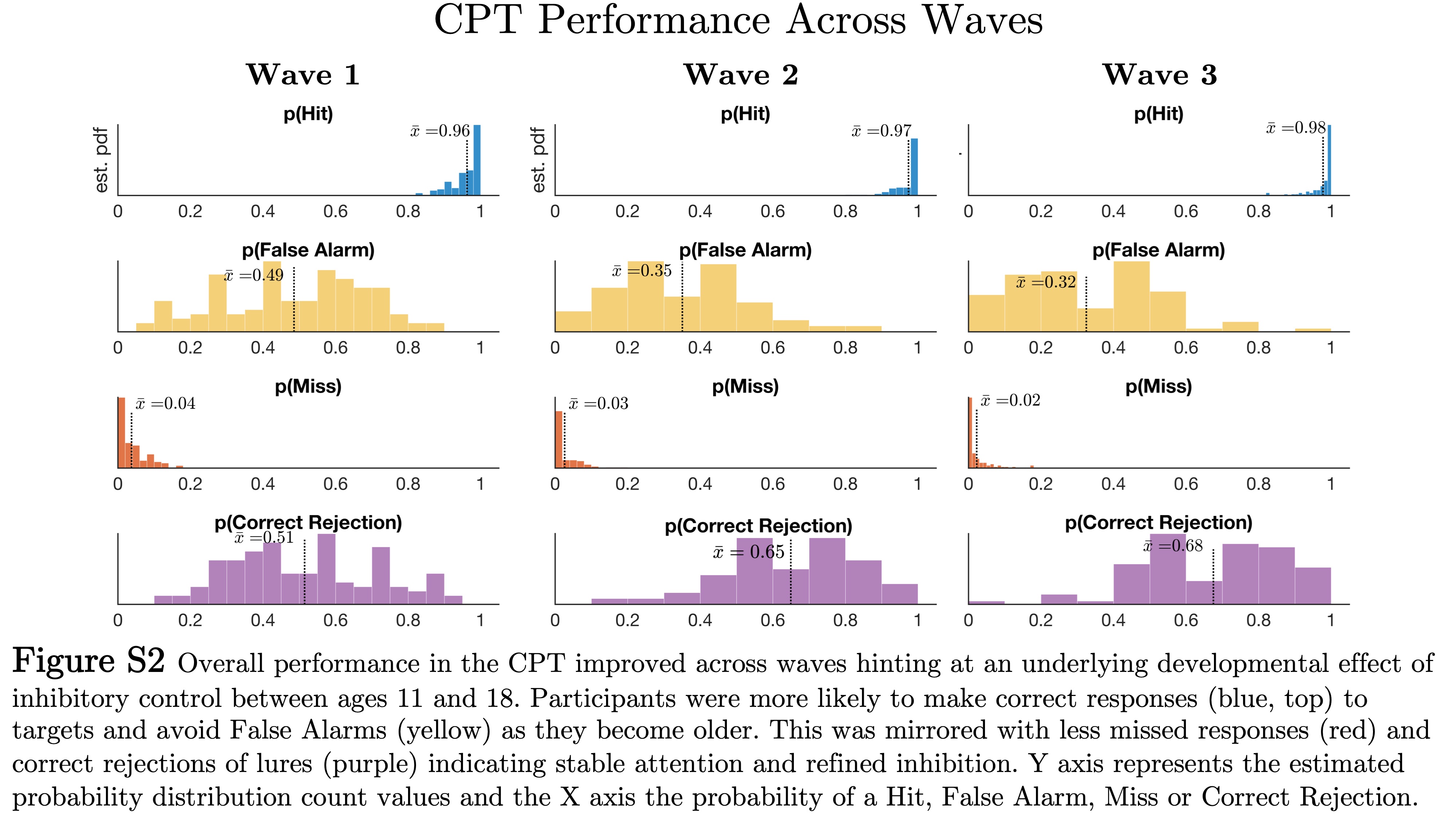

Supplement: Supplementary file 2 [file Image_2.jpeg]

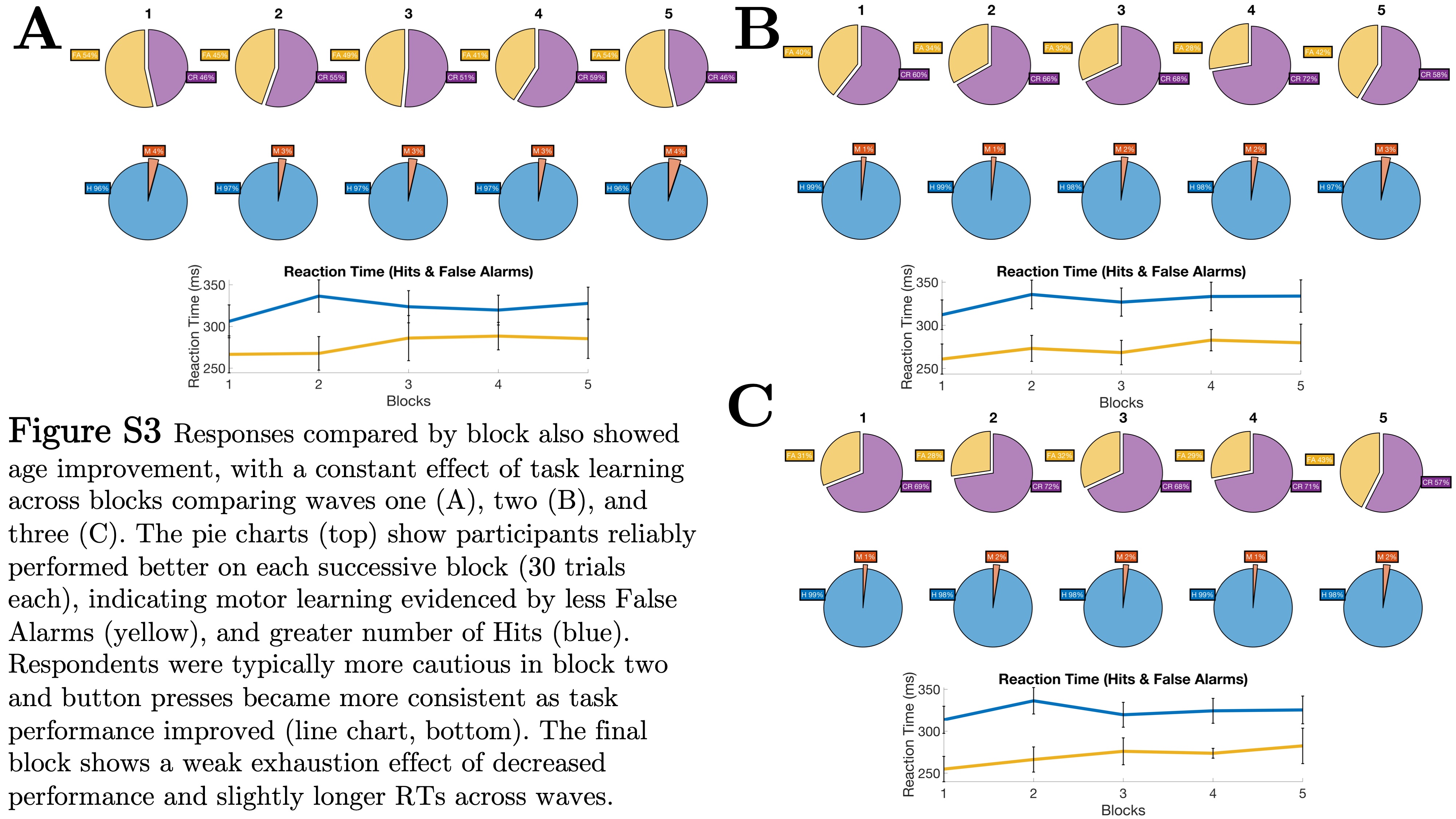

Supplement: Supplementary file 3 [file Image_3.jpeg]

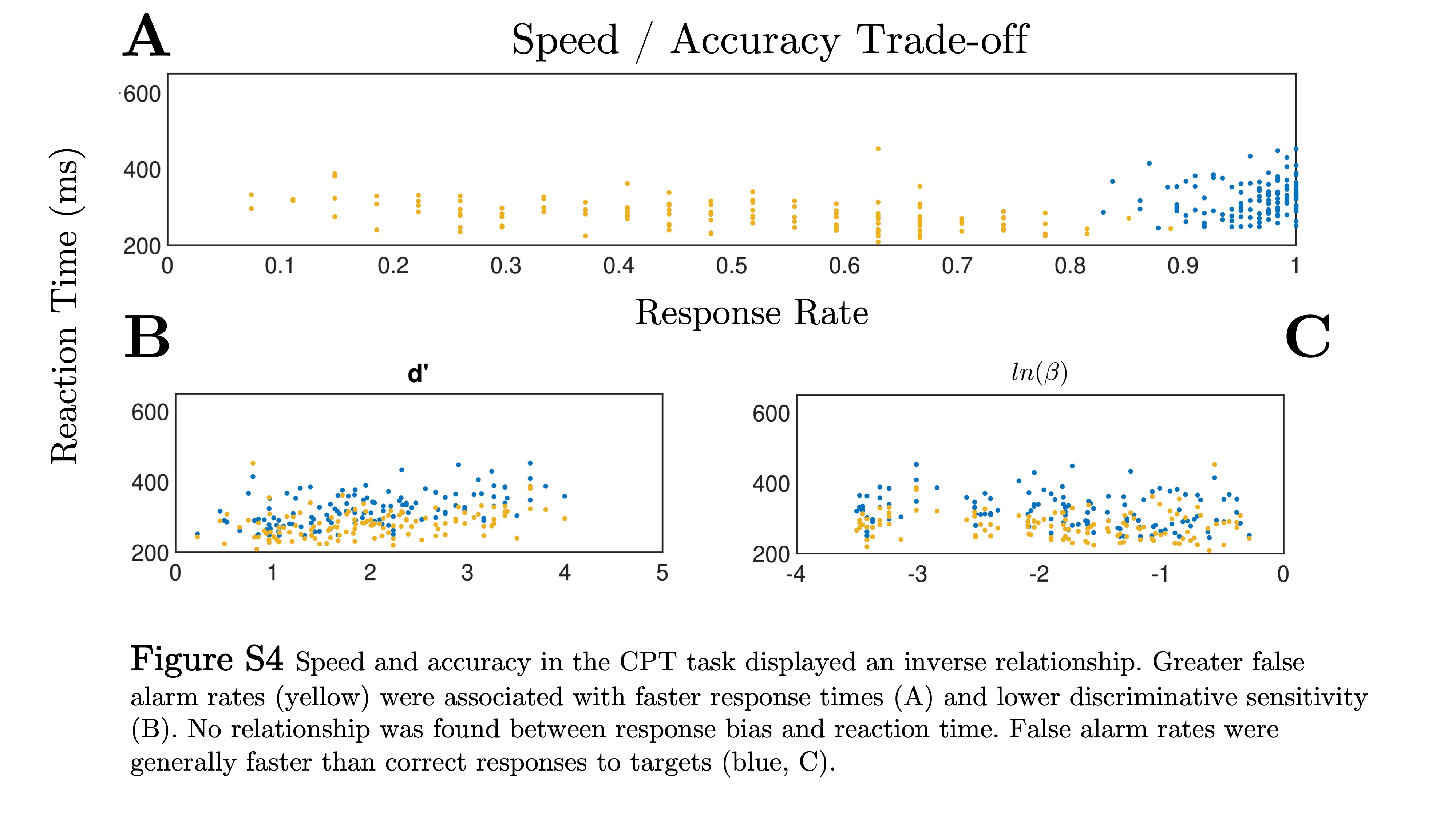

Supplement: Supplementary file 4 [file Image_4.jpeg]
